# The importance of overweight in COVID-19: A retrospective analysis in a single center of Wuhan, China

**DOI:** 10.1097/MD.0000000000022766

**Published:** 2020-10-23

**Authors:** Xinrui Rao, Chuangyan Wu, Sihua Wang, Song Tong, Geng Wang, Gang Wu, Rui Zhou

**Affiliations:** a Cancer center; b Department of Thoracic Surgery; c Department of Gastrointestinal Surgery, Union Hospital, Tongji Medical College, Huazhong University of Science and Technology, Wuhan, China.

**Keywords:** body mass index, China, COVID-19, overweight, severe pneumonia

## Abstract

The aim of this study was to evaluate the association between overweight and severity, drug response, and clinical outcomes of novel coronavirus disease 2019 (COVID-19).

In this retrospective cohort study, we reviewed medical records of 240 COVID-19 patients admitted to Union Hospital in Wuhan, China, between December 24, 2019, and March 25, 2020. Physical, clinical, laboratory, radiological characteristics, treatment, and outcome data were abstracted. Patients who were obese [body mass index (BMI) ≥28 kg/m^2^], underweight (BMI < 18.5 kg/m^2^), under 18 years old, pregnant, or still in hospital were excluded. Disease severity was classified as moderate or severe pneumonia based on the World Health Organization interim guidance. Overweight was defined as BMI ≥24 kg/m^2^ and <28 kg/m^2^. Patients were followed for discharge or death through April 10, 2020. We used logistic regression models to identify risk factors for severe disease, Cox proportional hazard models to explore associations between medications and patient outcomes (discharge or in-hospital death), and Kaplan–Meier survival curves and Cox regression models to evaluate risk factors for in-hospital death.

One-half of patients (120, 50.0%) had severe pneumonia, while nearly one-half (114, 47.5%) were overweight. Among patients over 45 years old, overweight patients had significantly lower rates of fatigue, higher rates of headache, and higher median C-reactive protein levels. Patients under 45 years old had higher rates of cough and myalgia and higher proportions of increased alanine aminotransferase and lactic dehydrogenase, as well as more pulmonary lobes involved in the pneumonia revealed by chest computed tomography scans. Overweight patients were at higher risk of developing severe pneumonia. Although weight was not a risk factor for in-hospital death, overweight patients showed different responses to medications compared with normal weight patients. Intravenous interferon-α, intravenous glucocorticoids, and antifungal drugs were associated with reduced mortality in overweight patients. Intravenous immunoglobulin, oseltamivir, and ribavirin were associated with reduced mortality in normal weight patients.

Overweight is a worldwide health problem. We found overweight to be related to the COVID-19 severity but not to in-hospital death. Clinicians should be aware that overweight COVID-19 patients require increased attention for different clinical features and treatment response.

## 1 Introduction

The year 2020 has seen novel coronavirus disease 2019 (COVID-19) spread rapidly worldwide. As of August 9, 2020, the COVID-19 pandemic has registered 19,714,788 cases worldwide with numbers increasing by 30,000 each day. Over 724,856 people are known to have died, and countless medical resources have been expended in control and treatment. Recognition of risk factors for severe COVID-19-related pneumonia, as well as predictive factors of treatment response, is of vital importance for the appropriate treatment and effective allocation of medical resources. However, definitive clinical trial data identifying safe and effective treatments for COVID-19 are not yet available.

Excess weight is one of the major chronic diseases that endanger human health. The global prevalence of obesity has increased substantially over the past 40 years—from less than 1% to 6% to 8% among minors, from 3% to 11% among men, and from 6% to 15% among women.^[1]^ As a major risk factor for noncommunicable diseases, obesity is associated with a decreased life expectancy of an estimated 5 to 20 years, depending on the severity of the condition and comorbid disorders.^[2]^ Overweight and obesity are present in one-fifth of intensive care unit (ICU) patients and are also associated with increased mortality and morbidity from infectious diseases.^[3–5]^ Of note, obesity has been shown to have affected the disease course and increased mortality rates in the influenza H1N1 pandemic^[6,7]^ and has been associated with impaired immune response to influenza vaccination in humans.^[8]^

Recent studies have linked obesity to COVID-19. One study reported that obesity in patients younger than 60 years is a risk factor for COVID-19-related hospital admission.^[9]^ Another study showed a high frequency of obesity among patients admitted to intensive care for severe acute respiratory syndrome coronavirus 2 (SARS-CoV-2).^[10]^ Among over 5000 patients with COVID-19 at an academic health system in New York City, obesity was an independent risk factor for hospitalization.^[11]^

In China, overweight is more prevalent than obesity and is present in over 30% of the adult population,^[12]^ and rates are continuing to rise. However, although it is generally accepted that comorbidities such as diabetes mellitus and cardiovascular disease are associated with the severity of COVID-19,^[13,14]^ overweight and obesity—main risk factors for these comorbidities^[15,16]^—have not been studied in detail. It is also unclear if there is an independent impact of overweight on the clinical course and prognosis of the disease. Finally, an area particularly deficient in information is the relationship between excess weight and treatment efficacy. In this study, we analyzed clinical details of COVID-19 patients admitted to Union Hospital in Wuhan, China, the earliest hospital to treat COVID-19 patients with severe pneumonia, and to assess the role that overweight plays in the clinical course and prognosis of COVID-19.

## 2 Methods

### 2.1 Patients

For this retrospective cohort study, we reviewed the electronic medical records of 283 COVID-19 patients admitted to the infectious disease ward of the Union Hospital, Tongji Medical College, Huazhong University of Science and Technology from December 24, 2019, to March 25, 2020. Eligible patients had been diagnosed with COVID-19 upon hospital admission: throat-swab samples positive for SARS-CoV-2 RNA accompanied by related symptoms such as fever, cough, shortness of breath, or difficulty breathing. Patients were discharged from the hospital after an absence of fever for at least 3 days, substantial improvement in both lungs based on a computed tomography (CT) scan of the chest, clinical remission of respiratory symptoms, and 2 negative throat-swab samples obtained at least 24 hours apart. Clinical outcomes (discharge or death) were recorded through April 10, 2020. Patients who were under 18 years old, pregnant, or still in hospital were excluded. Obese patients [body mass index (BMI) ≥28 kg/m^2^] and underweight patients (BMI < 18.5 kg/m^2^) were also excluded because the number was too small to analyze.

### 2.2 Data collection

We reviewed clinical charts, nursing records, laboratory findings, and chest CT scans for all eligible patients. Data on patients’ symptoms and physical status were abstracted from their hospital admission records; blood tests and CT scan results were abstracted for the first result after admission. Two researchers independently reviewed the data collection forms to double-check the quality of data abstraction.

### 2.3 Variable definitions

Using the clinical information in the patients’ records, patients were divided into severe and moderate disease status according to the WHO interim guidance (May 2020),^[17]^ which assesses clinical signs of pneumonia. Using the WHO guidelines, weight at hospital admission was categorized by BMI as normal weight (18.5–23.9 kg/m^2^) and overweight (24.0–27.9 kg/m^2^).^[18]^ Fever was defined as an axillary temperature higher than 37.3°C. Lymphopenia was defined by a lymphocyte count <1.0 × 10^9^/L. Smoking history was defined as having smoked more than one cigarette per day over the past 6 months. Alcohol intake was defined as drinking alcohol 4 or more times in a week, with more than 1 ounce of spirits (9 g of pure alcohol) each time. Respiratory supportive treatment included oxygen inhalation, noninvasive ventilation, invasive mechanical ventilation, and extracorporeal membrane oxygenation (ECMO).

### 2.4 Statistical analysis

All statistical analyses were conducted using the SPSS statistical software, version 23.0 (IBM Corp., Armonk, NY). To better present the extremum of time information, data were expressed as median (range) if they were time information or median (interquartile range, IQR) if they were not for continuous variables, and count (percentage) for categorical variables. Categorical variables between groups were compared using the χ^2^ test or Fisher exact test, and continuous variables were analyzed using the Student *t* test, analysis of variance (ANOVA), or Mann–Whitney *U* test, as appropriate. A *P* value < .05 was considered statistically significant.

We used univariate and multivariable logistic regression models to explore risk factors associated with disease severity, with moderate disease being the reference level. Nine variables were chosen for multivariable logistic regression analysis adjusted for smoking and drinking status based on their significance in the univariate regression analysis and clinical constraints. We excluded variables from the logistic regressions if the number of events was too small (≤3) to calculate odds ratios (ORs). Kaplan–Meier survival curves were plotted to reveal the association between severity and in-hospital death.

To better assess the age-specific clinical features of overweight patients, analyses were stratified by age. The dividing age of 45 years was chosen based on the age distributions of our sample (median age = 48) and the WHO classification for the middle age and the elderly.^[19]^

We further explored the association between medications and patient outcomes (discharge or death) using the Cox proportional hazards analysis. All models were adjusted for gender, age, comorbidities, smoking and drinking status, disease severity, and respiratory supportive treatment. All categories of medication recorded in the patients’ records, including antiviral agents, intravenous glucocorticoids, intravenous (standard) immunoglobulin, antifungal drugs, and antibiotics, were included in the models.

Kaplan–Meier survival curves were plotted to explore the association between weight and in-hospital death, with differences between the overweight and normal weight curves compared using the log-rank test. Multivariable Cox regression models were used to evaluate independent risk factors for death during hospitalization. The variables included in these models were chosen on the basis of previous findings and clinical constraints. For example, comorbidities including cardiac diseases and diabetes have been shown to correlate with poorer prognosis of COVID-19,^[13,14]^ and blood levels of d-dimer have been found to be higher in fatal cases.^[20]^ We excluded variables from the Cox regression analysis if the number of events (≤3) was too small to calculate hazard ratios.

## 3 Results

### 3.1 Patient characteristics

**Figure 1:**
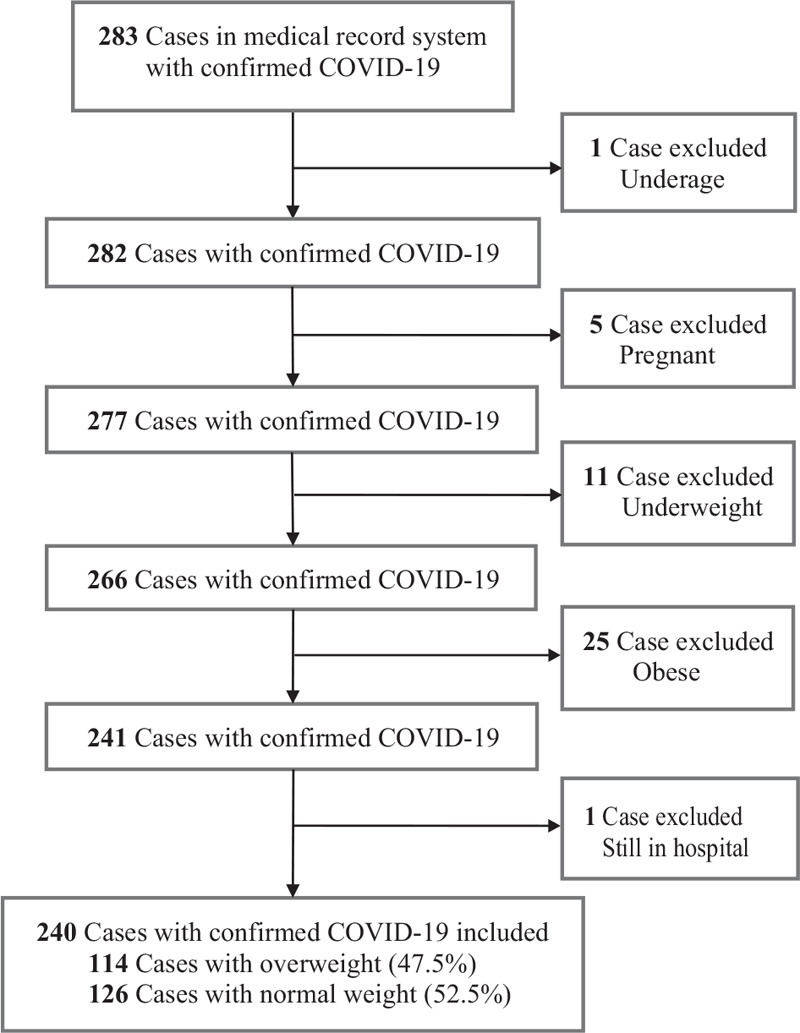
Flowchart of patient recruitment.

The final study population included 240 patients hospitalized with confirmed COVID-19, 120 of whom (50.0%) had severe pneumonia according to the WHO interim guidance (Fig. 1).^[17]^ The median (range) follow-up time was 13 (1–83) days. During follow-up, a total of 25 patients (10.4%) died and 215 patients (86.3%) were discharged (Table 1 ). The baseline characteristics of all patients are presented in Table 1 . Overall, the median age was 48 (23–87) years; 129 (53.8%) of the patients were female. The median BMI (IQR) was 23.9 (21.91–25.95) kg/m^2^. Of all patients, 126 (52.5%) were normal weight and 114 (47.5%) were overweight. The most common symptom at onset of illness was fever [211 (90.6%)], followed by cough [124 (58.2%)] and fatigue [101 (47.4%)]. Cardiovascular diseases [43 (17.9%)] and diabetes [23 (9.6%)] were the most common comorbidities.

**Table 1:**
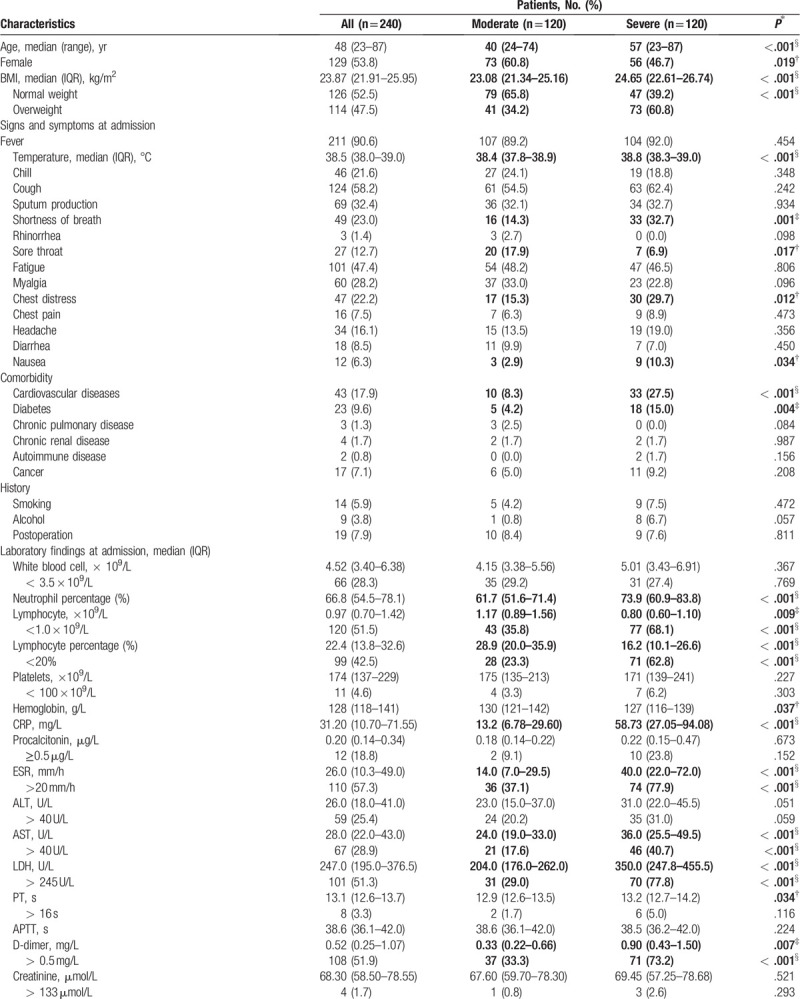
Overall clinical characteristics of 240 patients with COVID-19.

**Table 1 (Continued):**
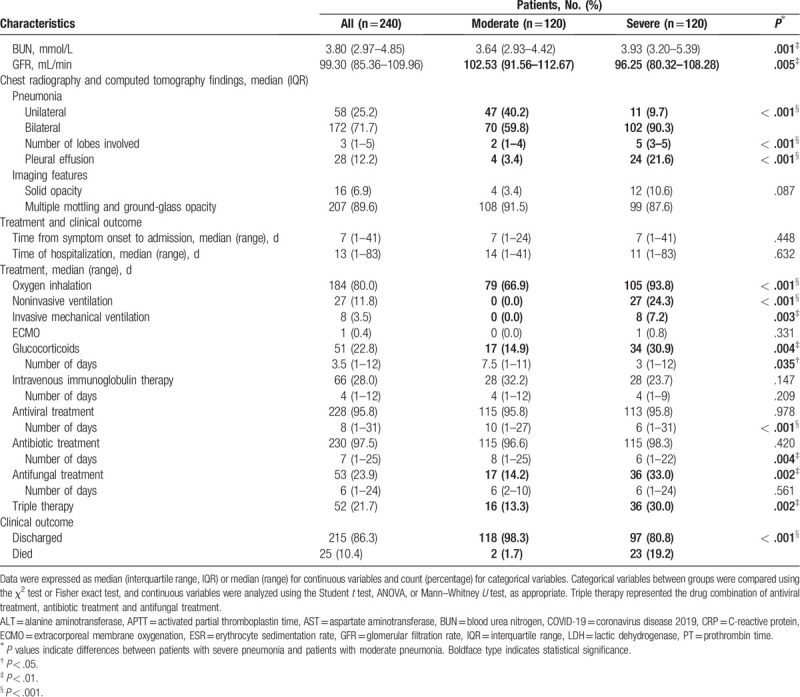
Overall clinical characteristics of 240 patients with COVID-19.

### 3.2 Factors associated with the severity of pneumonia

Patients with severe disease had significantly higher BMI levels and were more likely to be overweight [73 (60.8%) vs 41 (34.2%), *P* < .001]. They were also more likely to show symptoms such as shortness of breath and chest distress, and to have higher rates of cardiovascular diseases and diabetes at admission (Table 1 ).

The blood counts of patients with severe pneumonia showed more severe lymphopenia than did those of patients with moderate pneumonia. Lymphocyte counts were considerably lower in the severe group, while C-reactive protein (CRP) levels (CRP) and erythrocyte sedimentation rates (ESRs) were significantly higher in the severe group (Table 1 ).

CT scans showed high levels of multiple mottling and ground-glass opacity in all patients; the difference by disease status was not significant. However, patients with severe pneumonia had greater involvement of the pulmonary lobes and a higher incidence of pleural effusion (Table 1 ).

A higher proportion of patients with severe pneumonia received glucocorticoid and antifungal drug treatment compared with patients with moderate pneumonia. Furthermore, the usage rate of combined antiviral, antibiotic, and antifungal treatments was significantly higher in the severe group (Table 1 ), likely reflecting more serious symptoms and coinfections accompanying severe disease. The mortality rate was higher among patients with severe pneumonia compared with patients with moderate pneumonia as shown in Table 1  and the Kaplan--Meier survival curves in Supplementary Figure 1.

### 3.3 Associations of overweight with severity of pneumonia

**Figure 2:**
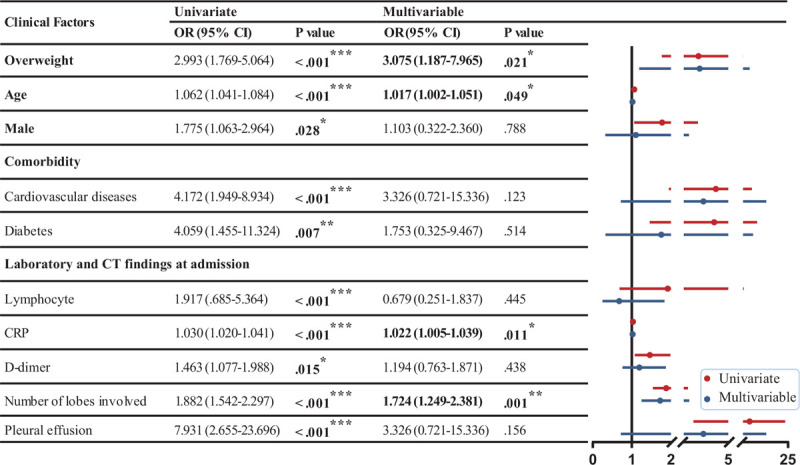
Logistic regression analysis for the risk of severe pneumonia. Adjusted for smoking and drinking status, odds ratio (95% confidence interval) are shown for the risk factors associated with severe pneumonia. Boldface type indicates statistical significance. CI = confidence interval; CRP = C-reactive protein; OR = odds ratio. ^∗^*P* < .05, ^†^*P* < .01, ^‡^*P* < .001.

Figure 2 shows univariate and multivariable logistic regression results for the risk of severe pneumonia. We found that overweight patients were more likely to develop severe pneumonia than were normal-weight patients [73 (64.0%) vs 47 (37.3%), *P* < .001]. Male sex, cardiovascular diseases, and diabetes showed significant positive associations with severe disease but were not independent risk factors in the multivariable analysis model. Both the univariate and multivariable logistic regression analysis indicated that older age [OR, 95% confidence interval (95% CI), multivariable: 1.017 (1.002–1.051), *P* = .049] and overweight [OR (95% CI), multivariable: 3.075 (1.187–7.965), *P* = .021] were independent risk factors for severe pneumonia (Fig. 2).

### 3.4 Age-specific clinical features of overweight patients

As age has been shown to be an independent risk factor for the severity of COVID-19 in the literature as well as in our regression models, we examined our results stratified by age. Among patients over 45 years of age, overweight patients were significantly less likely to report feeling fatigue but were more likely to develop severe pneumonia and to report headaches. For patients under 45 years of age, higher rates of cough and myalgia were reported (Table 2 ).

**Table 2:**
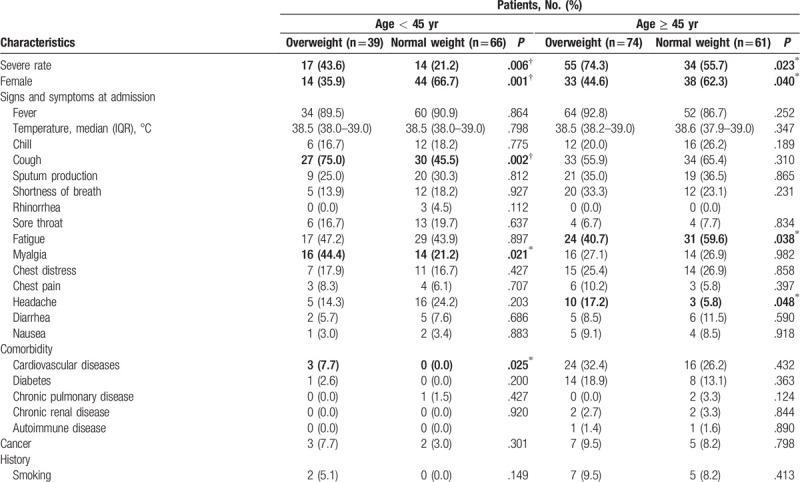
Age-specific clinical features of overweight patients with COVID-19.

**Table 2 (Continued):**
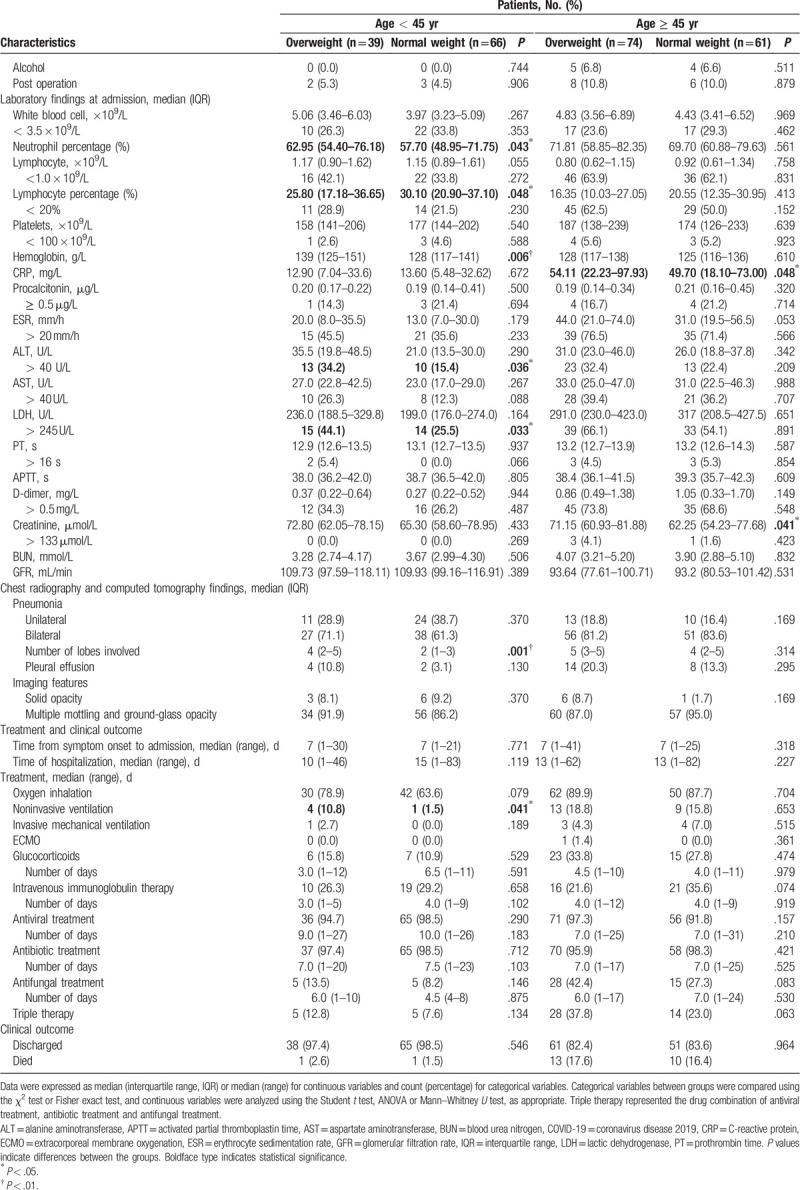
Age-specific clinical features of overweight patients with COVID-19.

Laboratory findings indicated that among patients over 45 years of age, overweight patients had significantly higher median CRP levels than did normal-weight patients (Table 2 ). Among patients under 45 years of age, overweight patients showed higher proportion of liver impairment [alanine aminotransferase (ALT) > 40 U/L]. Elevated levels of lactic dehydrogenase (LDH > 245 U/L) were also observed, probably related to the myalgia reported by patients. More pulmonary lobes were involved in the pneumonia as revealed by the chest CT scans among patients under 45 years of age (Table 2 ).

### 3.5 Response to medical treatment by patient weight

Among all treatments, the proportion of antibiotic treatment was the highest [230 (97.5%)], followed by antiviral therapy [228 (95.8%)] and intravenous immunoglobulin therapy [66 (28.0%)] (Tables 1 and 2 ). There were no significant differences in the administration of medications between the overweight and normal-weight patients. When stratified by age, we found that overweight patients under 45 years of age were more likely to have received noninvasive ventilation, which may be due to their disease having progressed to the severe level (Table 2 ).

**Figure 3:**
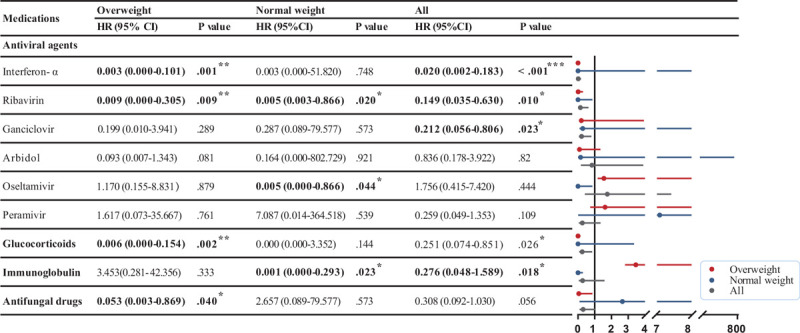
Multivariable Cox proportional hazards analysis for the association between medications and patient outcomes. Adjusted for gender, age, comorbidities, smoking and drinking status, disease severity, and respiratory supportive treatment. Boldface type indicates statistical significance. CI = confidence interval; HR = hazard ratio. ^∗^*P* < .05, ^†^*P* < .01, ^‡^*P* < .001.

We used Cox proportional hazard models to explore the independent associations of medications with outcomes for overweight patients and normal-weight patients (Fig. 3). Intravenous glucocorticoids [hazard ratio (HR) (95% CI), 0.006 (0.000–0.154), *P* = .002] and antifungal drugs [HR (95% CI), 0.053 (0.003–0.869), *P* = .040] were associated with reduced mortality. Among the antiviral agents, intravenous interferon-α [HR (95% CI), 0.003 (0.000–0.101), *P* = .002] and ribavirin [HR (95% CI), 0.009 (0.000–0.305), *P* = .009] were associated with reduced mortality. In the normal-weight group, intravenous immunoglobulin [HR (95% CI), 0.001 (0.000–0.293), *P* = .02], oseltamivir [HR (95% CI), 0.005 (0.000–0.866), *P* = .04], and ribavirin [HR (95% CI), 0.005 (0.003–0.866), *P* = .02] were related to reduced mortality. These results suggest differences in drug response between overweight and normal-weight patients (Fig. 3).

### 3.6 Overweight as a risk factor for in-hospital death

**Figure 4:**
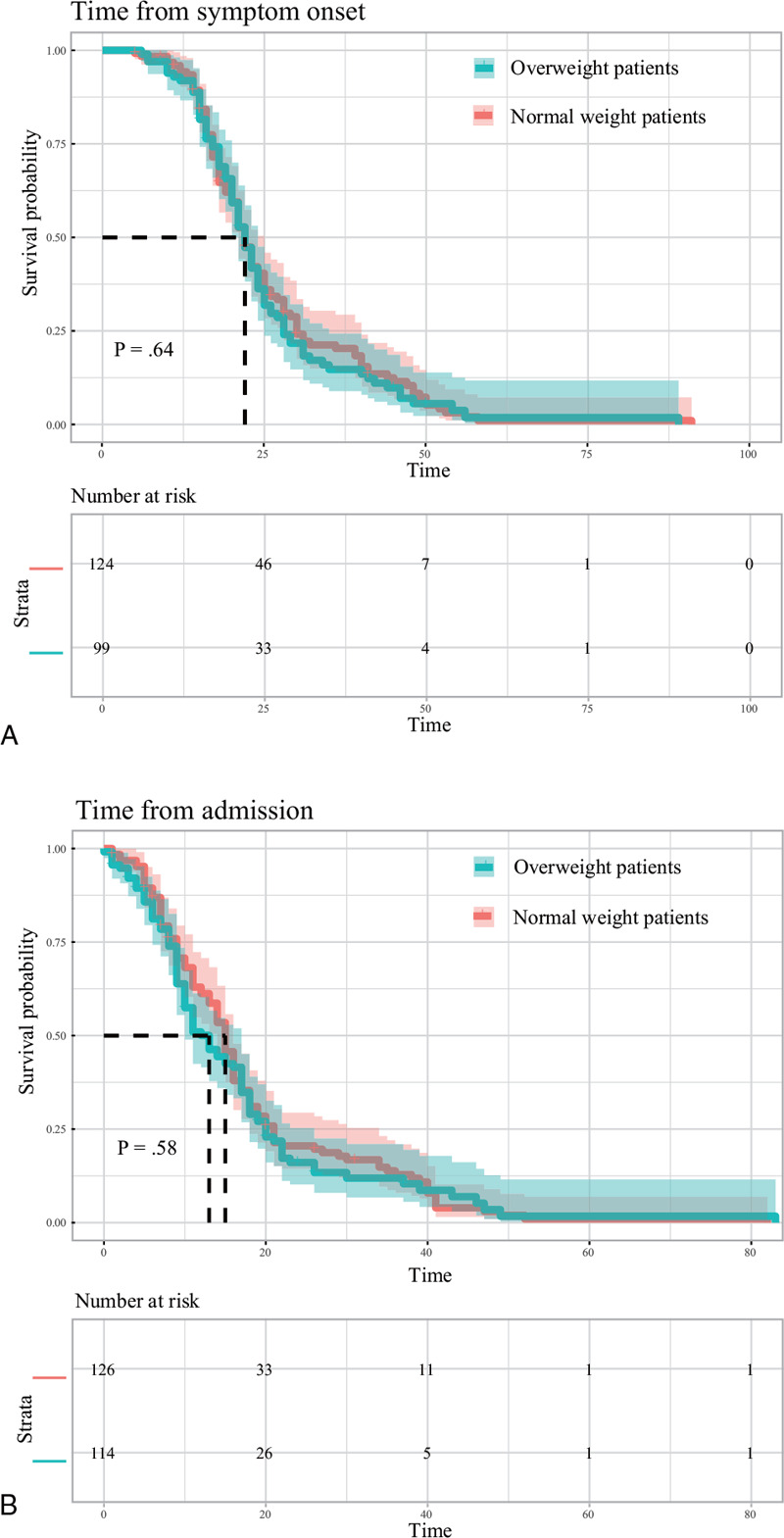
Survival analysis of mortality during hospitalization by patient weight. Kaplan–Meier survival curves for mortality during the time from symptom onset (A) and admission (B).

**Figure 5:**
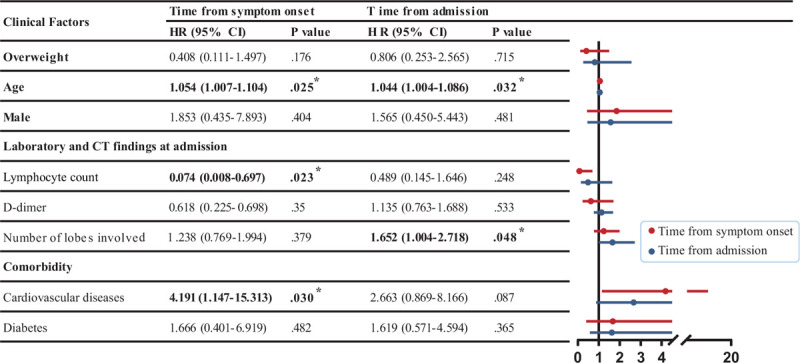
Multivariable Cox proportional hazards analysis for the risk of mortality. Hazard ratio (95% confidence interval) are shown for the risk factors associated with in-hospital death. CI = confidence interval; HR = hazard ratio. Boldface type indicates statistical significance. ^∗^*P* < .05, ^†^*P* < .01, ^‡^*P* < .001.

As our data found that overweight was an independent risk factor for the severity level of COVID-19, we hypothesized that overweight would also be associated with prognosis. However, survival curves did not show significant differences in outcomes between the two groups (Fig. 4A and B). Furthermore, the Cox proportional hazards analysis did not find overweight to be a significant risk factor for mortality, based either on duration from symptom onset to time of death or from hospital admission to time of death (Fig. 5).

## 4 Discussion

To our knowledge, this is the first report of weight-specific data of COVID-19 patients. Despite a higher proportion of severe disease, we did not find significant differences in outcomes (discharge or death) between overweight and normal weight patients. There were, however, statistically significant differences by patient weight for symptoms, laboratory results and chest CT scans at hospital admission, severity of pneumonia, and treatment response. First, we found overweight to be an independent risk factor for severity of COVID-19. People with excess weight are more likely to have comorbidities, including metabolic diseases, cardiovascular diseases, and some types of cancer.^[2]^ However, after adjustment for comorbidities and other cofounding factors, overweight continued to show a significant relationship with the increased risk of severe illness. More studies are needed to further explore how excess weight affects the progression of the disease independently from comorbid conditions. Second, overweight patients were more likely to report symptoms of headache and myalgia than were normal weight patients. Their laboratory findings presented lower percentages of lymphocytes and higher inflammatory biomarkers (e.g., CRP), which have been considered as indicators of severity.^[21]^ Similar to previous reports, we also found that overweight COVID-19 patients showed higher levels of ALT, implying liver impairment. However, impairment of liver function does not appear to be a prominent feature of COVID-19 and may not have serious clinical consequences.^[22]^ Third, CT results of overweight patients under 45 years of age revealed greater involvement of pulmonary lobes, which has also been reported to be an indicator of severe SARS-CoV-2 pneumonia.^[23]^

Over the past decades, studies have examined the role of inflammation in the connection between obesity and chronic diseases.^[24]^ The inflammatory response triggered by obesity involves many components of the classical inflammatory response to pathogens and includes systemic increases in circulating inflammatory cytokines and acute phase proteins (e.g., CRP), recruitment of leukocytes to inflamed tissues, activation of tissue leukocytes, and generation of reparative tissue responses.^[24]^ In this regard, low-grade inflammation may be involved in the progression of the COVID-19 in overweight and obese patients.

We also explored the association between medication type and outcomes by patient weight, finding that overweight and normal-weight patients presented different responses to the various treatments. Ribavirin, a guanosine analogue, is an antiviral compound used to treat several virus infections, including respiratory syncytial virus, hepatitis C virus, and some viral hemorrhagic fevers^[25]^; we found a relationship with lower mortality in both normal-weight and overweight patients. A molecular modeling study reported that ribavirin targets SARS-CoV-2 RNA-dependent RNA polymerase (RdRp).^[26]^ Although the efficacy of ribavirin in COVID-19 remains controversial, it was widely used in the early stages of the outbreak due to its availability and low cost. A phase 2 trial on COVID-19 treatments at the University of Hong Kong has shown that the triple combination of interferon beta-1b, lopinavir-ritonavir, and ribavirin reduced the median time from the start of the study treatment to a negative nasopharyngeal swab.^[27]^ Multiple trials are still underway to evaluate the specific role of ribavirin in the treatment of COVID-19.

Interferon-α, a broad-spectrum antiviral agent that interacts with toll-like receptors and which inhibits viral replication, was negatively correlated with the mortality of overweight patients in our study. A current clinical trial (ChiCTR2000029387) is evaluating its efficacy and safety when used in combination with ribavirin, lopinavir/ritonavir (LPV/RTV), or ribavirin as well as LPV/RTV for SARS-CoV-2 infection.^[28]^ Respiratory failure caused by severe cytokine and chemokine storms has been reported to be associated with mortality in COVID-19.^[29]^ Glucocorticoids are important in modulating the immune system when it is in a hyperinflammatory state. Our results suggest that the use of glucocorticoids in overweight patients is associated with favorable outcomes. The underlying mechanism may involve the control of immunopathological lung damage in severe pneumonia.

Intravenous immunoglobulin, another agent that can interrupt the storm of inflammatory cytokines, showed negative associations with mortality in normal-weight patients. Studies have shown their efficacy in the treatment of patients with severe inflammatory complaints related to SARS-CoV-2 infections.^[30]^ Although no consensus has been reached on the use of glucocorticoids and intravenous immunoglobulins due to possible adverse interactions, they are both effective in managing manifestations of severe inflammatory disorders such as high fever and respiratory failure caused by pneumonia. Thus, careful use of these medications, with attention to timing, may be beneficial. We further found that antifungal drugs were negatively related to mortality in overweight patients. Although there is no evidence supporting the efficacy of antifungal drugs on the SARS-CoV-2 virus, the use of these drugs was mainly based on coinfections. The observed relationship to improved outcomes of overweight patients may thus be due to their impact on fungal infections, reducing the severity of pneumonia.

As our data were collected during the early stages of the COVID-19 outbreak, treatment methods observed in our study differed from current practices around the world. For example, the lower usage of mechanical ventilation and the higher usage of antibiotics were observed in our study, which may be due to shortages of medical drugs and equipment and a lack of knowledge of the disease at that time. The type and dose of medications used relied mainly on doctors’ experiences with viral pneumonia. For instance, we found a higher usage of the antiviral agent oseltamivir, which was likely related to the overlap of the early COVID-19 outbreak with the peak influenza season. However, as more evidence emerges, significant changes have taken place in the COVID-19 treatment. A randomized controlled trial suggested that remdesivir significantly reduced time to recovery compared with placebo,^[31]^ and it has been approved by the Food and Drug Administration for emergency use with COVID-19. However, although widely used in the past as antivirals, current COVID-19 treatment guidelines from National Institute of Health (NIH) do not recommend the use of chloroquine or hydroxychloroquine to treat COVID-19 other than in clinical trials.^[32]^ A large, retrospective, observational study evaluating the use of hydroxychloroquine has shown no evidence of benefit in patients with COVID-19.^[33]^ Currently, there are over 450 ongoing registered clinical trials investigating the efficacy of new antiviral drugs, immune-based therapies, and vaccines.^[34]^ This research is likely to provide important evidence for COVID-19 treatments in the near future.

Studies to date have partially described the link between obesity and COVID-19. This is the first comprehensive analysis, including clinical data and follow-up, of the impact of overweight in the progression of COVID-19. However, there are limitations to our study. First, it is a descriptive study from a single center early in the early pandemic with a relatively small sample size. Second, all predictive data were collected on the time of hospital admission, and it was not possible to monitor the changes in the clinical parameters. Additional, comprehensive, prospective, clinical and pathological studies are needed.

## 5 Conclusion

Our study found that patient weight was significantly associated with the efficacy of COVID-19 treatment. Given the status of overweight and obesity as a global health problem, our results have important implications for clinicians on the progression of COVID-19 and for appropriate treatment. As the pandemic continues, overweight patients will require increased attention based on their different clinical features and, importantly, differential responses to treatment. Worldwide, increased emphasis on weight management is also an important strategy to reduce the impact of COVID-19.

## Author contributions

**Data curation:** Chuangyan Wu.

**Formal analysis:** Xinrui Rao, Rui Zhou.

**Investigation:** Xinrui Rao, Rui Zhou.

**Methodology:** Chuangyan Wu, Song Tong.

**Resources:** Chuangyan Wu, Sihua Wang, Song Tong.

**Software:** Xinrui Rao.

**Supervision:** Rui Zhou, Gang Wu.

**Validation:** Chuangyan Wu, Sihua Wang, Geng Wang.

**Writing – original draft:** Xinrui Rao.

**Writing – review & editing:** Chuangyan Wu, Sihua Wang, Song Tong, Geng Wang, Rui Zhou.

## Supplementary Material

Supplemental Digital Content

## References

[1] Jaacks LM Vandevijvere S Pan A The obesity transition: stages of the global epidemic. Lancet Diabetes Endocrinol 2019;7:231–40. 30704950 10.1016/S2213-8587(19)30026-9 PMC7360432

[2] Bluher M Obesity: global epidemiology and pathogenesis. Nat Rev Endocrinol 2019;15:288–98. 30814686 10.1038/s41574-019-0176-8

[3] Oz HS Nutrients, Infectious and Inflammatory Diseases. Nutrients 2017;9:1085–93. 10.3390/nu9101085 PMC5691702 28973995

[4] Huttunen R Syrjanen J Obesity and the risk and outcome of infection. Int J Obes (Lond) 2013;37:333–40. 22546772 10.1038/ijo.2012.62

[5] Schetz M De Jong A Deane AM Obesity in the critically ill: a narrative review. Intensive Care Med 2019;45:757–69. 30888440 10.1007/s00134-019-05594-1

[6] Louie JK Acosta M Samuel MC A novel risk factor for a novel virus: obesity and 2009 pandemic influenza A (H1N1). Clin Infect Dis 2011;52:301–12. 21208911 10.1093/cid/ciq152

[7] Fuhrman C Bonmarin I Bitar D Adult intensive-care patients with 2009 pandemic influenza A (H1N1) infection. Epidemiol Infect 2011;139:1202–9. 20974021 10.1017/S0950268810002414

[8] Sheridan PA Paich HA Handy J Obesity is associated with impaired immune response to influenza vaccination in humans. Int J Obes (Lond) 2012;36:1072–7. 22024641 10.1038/ijo.2011.208 PMC3270113

[9] Lighter J Phillips M Hochman S Obesity in patients younger than 60 years is a risk factor for Covid-19 hospital admission. Clin Infect Dis 2020;71:896–7. 32271368 10.1093/cid/ciaa415 PMC7184372

[10] Simonnet A Chetboun M Poissy J High prevalence of obesity in severe acute respiratory syndrome coronavirus-2 (SARS-CoV-2) requiring invasive mechanical ventilation. Obesity (Silver Spring) 2020;28:1195–9. 32271993 10.1002/oby.22831 PMC7262326

[11] Petrilli CM Jones SA Yang J Factors associated with hospital admission and critical illness among 5279 people with coronavirus disease 2019 in New York City: prospective cohort study. BMJ 2020;369:m1966. 32444366 10.1136/bmj.m1966 PMC7243801

[12] Chen Y Peng Q Yang Y The prevalence and increasing trends of overweight, general obesity, and abdominal obesity among Chinese adults: a repeated cross-sectional study. BMC Public Health 2019;19:1293. 31615464 10.1186/s12889-019-7633-0 PMC6794823

[13] Shi S Qin M Shen B Association of cardiac injury with mortality in hospitalized patients with COVID-19 in Wuhan, China. JAMA Cardiol 2020;5:802–10. 32211816 10.1001/jamacardio.2020.0950 PMC7097841

[14] Guo W Li M Dong Y Diabetes is a risk factor for the progression and prognosis of COVID-19. Diabetes Metab Res Rev 2020;e3319 Epub ahead of print. 32233013 10.1002/dmrr.3319 PMC7228407

[15] Lee CM Colagiuri S Ezzati M The burden of cardiovascular disease associated with high body mass index in the Asia-Pacific region. Obes Rev 2011;12:e454–9. 21366838 10.1111/j.1467-789X.2010.00849.x

[16] Chobot A Gorowska-Kowolik K Sokolowska M Obesity and diabetes: not only a simple link between two epidemics. Diabetes Metab Res Rev 2018;34:e3042. 29931823 10.1002/dmrr.3042 PMC6220876

[17] World Health Organization. Clinical Management of COVID-19. 2020 Available at: https://www.who.int/publications-detail/clinical-management-of-severe-acute-respiratory-infection-when-novel-coronavirus-(ncov)-infection-is-suspected. [Accessed May 27, 2020]. 35917394

[18] WHO Expert Consultation. Appropriate body-mass index for Asian populations and its implications for policy and intervention strategies. Lancet 2004;363:157–63. 14726171 10.1016/S0140-6736(03)15268-3

[19] World Health Organization. World Health Statistics 2020: Monitoring health for the SDGs. 2020; https://www.who.int/gho/publications/world_health_statistics/2020/en/ 2020

[20] Huang C Wang Y Li X Clinical features of patients infected with 2019 novel coronavirus in Wuhan, China. Lancet 2020;395:497–506. 31986264 10.1016/S0140-6736(20)30183-5 PMC7159299

[21] Terpos E Ntanasis-Stathopoulos I Elalamy I Hematological findings and complications of COVID-19. Am J Hematol 2020;95:834–47. 32282949 10.1002/ajh.25829 PMC7262337

[22] Zhang Y Zheng L Liu L Liver impairment in COVID-19 patients: a retrospective analysis of 115 cases from a single centre in Wuhan city, China. Liver Int 2020;40:2095–103. 32239796 10.1111/liv.14455

[23] Li M Chest CT features and their role in COVID-19. Radiol Infect Dis 2020;7:51–4. 32309528 10.1016/j.jrid.2020.04.001 PMC7162628

[24] Lumeng CN Saltiel AR Inflammatory links between obesity and metabolic disease. J Clin Invest 2011;121:2111–7. 21633179 10.1172/JCI57132 PMC3104776

[25] Wiersinga WJ Rhodes A Cheng AC Pathophysiology, Transmission, Diagnosis, and Treatment of Coronavirus Disease 2019 (COVID-19): A Review. JAMA 2020;324:782–93. 32648899 10.1001/jama.2020.12839

[26] Elfiky AA Anti-HCV, nucleotide inhibitors, repurposing against COVID-19. Life Sci 2020;248:117477. 32119961 10.1016/j.lfs.2020.117477 PMC7089605

[27] Hung IF-N Lung K-C Tso EY-K Triple combination of interferon beta-1b, lopinavir–ritonavir, and ribavirin in the treatment of patients admitted to hospital with COVID-19: an open-label, randomised, phase 2 trial. The Lancet 2020;395:1695–704. 10.1016/S0140-6736(20)31042-4 PMC7211500 32401715

[28] Chongqing Public Health Medical Center. ChiCTR2000029387. Comparative effectiveness and safety of ribavirin plus interferon-alpha, lopinavir/ritonavir plus interferon-alpha and ribavirin plus lopinavir/ritonavir plus interferon-alphain in patients with mild to moderate novel coronavirus pneumonia. 2020; http://www.chictr.org.cn/showproj.aspx?proj=48782. Accessed 29 January 2020.

[29] Alijotas-Reig J Esteve-Valverde E Belizna C Immunomodulatory therapy for the management of severe COVID-19. Beyond the anti-viral therapy: a comprehensive review. Autoimmun Rev 2020;19:102569. 32376394 10.1016/j.autrev.2020.102569 PMC7252146

[30] Lai ST Treatment of severe acute respiratory syndrome. Eur J Clin Microbiol Infect Dis 2005;24:583–91. 16172857 10.1007/s10096-005-0004-z PMC7088345

[31] Beigel JH Tomashek KM Dodd LE Remdesivir for the treatment of Covid-19: preliminary report. N Engl J Med 2020;[Epub ahead of print]. 10.1056/NEJMc2022236 32649078

[32] National Institute of Health. Coronavirus Disease 2019 (COVID-19) Treatment Guidelines. 2020. Available at: https://www.covid19treatmentguidelines.nih.gov/antiviral-therapy/chloroquine-or-hydroxychloroquine/. Accessed July 30, 2020.

[33] Geleris J Sun Y Platt J Observational study of hydroxychloroquine in hospitalized patients with Covid-19. N Engl J Med 2020;382:2411–8. 32379955 10.1056/NEJMoa2012410 PMC7224609

[34] Zhai P Ding Y Wu X The epidemiology, diagnosis and treatment of COVID-19. Int J Antimicrob Agents 2020;55:105955. 32234468 10.1016/j.ijantimicag.2020.105955 PMC7138178

