# Supplementary material for: The importance of overweight in COVID-19: A retrospective analysis in a single center of Wuhan, China
Source: Medicine (Baltimore). 2020 Oct 23;99(43):e22766. doi: 10.1097/MD.0000000000022766 (PMC7581045; doi:10.1097/MD.0000000000022766)

**Supplementary figure 1. Survival Analysis of Mortality During Hospitalization by Severity** Kaplan–Meier survival curves for mortality during the time from admission.


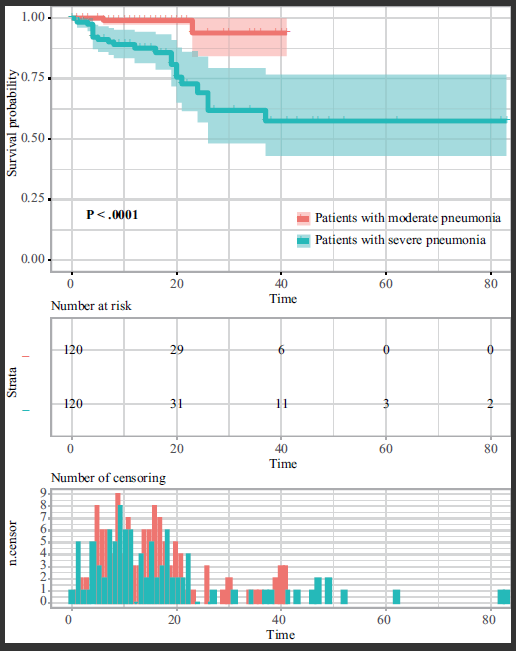

Supplement: Supplemental Digital Content [file medi-99-e22766-s001.doc]
